# Surgical and radiosurgical treatment of hypothalamic hamartoma: The Italian experience between 2011 and 2021

**DOI:** 10.1002/epi4.12989

**Published:** 2024-06-26

**Authors:** Michele Rizzi, Alessandro Consales, Irene Tramacere, Alessandro De Benedictis, Antonella Bua, Nicola Specchio, Luca De Palma, Erica Cognolato, Lino Nobili, Domenico Tortora, Carmen Barba, Marianna Pommella, Flavio Giordano, Chiara Pastori, Marcello Marchetti, Rita Garbelli, Mino Zucchelli, Matteo Martinoni, Lorenzo Ferri, Matia Martucci, Gianpiero Tamburrini, Federico Bianchi, Claudia Passamonti, Giancarlo Di Gennaro, Flavio Villani, Laura Tassi, Carlo Marras

**Affiliations:** ^1^ Functional Neurosurgery Unit, Department of Neurosurgery Fondazione IRCCS Istituto Neurologico Carlo Besta Milan Italy; ^2^ Neurosurgery Unit IRCCS Istituto Giannina Gaslini Genoa Italy; ^3^ Department of Research and Clinical Development, Scientific Directorate Fondazione IRCCS Istituto Neurologico Carlo Besta Milan Italy; ^4^ Neurosurgery Unit Bambino Gesù Children's Hospital IRCCS Rome Italy; ^5^ Clinical and Experimental Neurology Bambino Gesù Children's Hospital IRCCS Rome Italy; ^6^ Child Neuropsychiatry Unit IRCCS Istituto Giannina Gaslini Genoa Italy; ^7^ Department of Neurosciences, Rehabilitation, Ophthalmology, Genetics, Maternal and Child Health (DiNOGMI) University of Genoa Genoa Italy; ^8^ Neuroradiology Unit IRCCS Istituto Giannina Gaslini Genoa Italy; ^9^ Neuroscience Department Meyer Children's Hospital IRCCs Florence Italy; ^10^ Department of Neurosciences University of Florence Florence Italy; ^11^ Neurosurgery Unit Meyer Children's Hospital IRCCs Florence Italy; ^12^ Epilepsy Unit Fondazione IRCCS Istituto Neurologico Carlo Besta Milan Italy; ^13^ Unit of Radiotherapy, Department of Neurosurgery Fondazione IRCCS Istituto Neurologico Carlo Besta Milan Italy; ^14^ IRCCS Istituto Delle Scienze Neurologiche di Bologna Bologna Italy; ^15^ Department of Biomedical and Neuromotor Sciences University of Bologna Bologna Italy; ^16^ Diagnostic Neuroradiology Unit Fondazione Policlinico Gemelli IRCCS Rome Italy; ^17^ Università Cattolica del Sacro Cuore Rome Italy; ^18^ Pediatric Neurosurgery Fondazione Policlinico Gemelli IRCCS Rome Italy; ^19^ Psychology Unit Azienda Ospedaliero Universitaria Delle Marche Ancona Italy; ^20^ IRCCS Neuromed Pozzilli Italy; ^21^ Division of Clinical Neurophysiology and Epilepsy Center IRCCS Ospedale Policlinico San Martino Genoa Italy; ^22^ Claudio Munari Epilepsy Surgery Center ASST GOM Niguarda Milan Italy

**Keywords:** endoscopic neurosurgery, epilepsy surgery, hypothalamic hamartoma, laser interstitial thermal therapy, stereotactic radiosurgery

## Abstract

**Objective:**

To investigate the Italian experience on the surgical and radiosurgical treatment of drug‐resistant epilepsy due to hypothalamic hamartoma (HH) in the period 2011–2021 in six Italian epilepsy surgery centers, and to compare safety and efficacy profiles of the different techniques.

**Methods:**

We collected pseudo‐anonymized patient's data with at least 12 months of follow‐up. Surgical outcome was defined according to Engel classification of seizure outcome. Univariate analysis was performed to assess the risk of post‐operative seizures, categorized in dichotomous variable as favorable and unfavorable; explanatory variables were considered. Mann–Whitney or Chi‐squared test were used to assess the presence of an association between variables (*p* < 0.05).

**Results:**

Full presurgical and postoperative data about 42 patients from 6 epilepsy surgery centers were gathered. Engel class I was reached in the 65.8% and 66.6% of patients with gelastic and non‐gelastic seizures, respectively. Other than daily non‐gelastic seizures were associated with seizure freedom (*p* = 0.01), and the radiological type presented a trend toward significance (*p* = 0.12).

**Significance:**

Endoscopic disconnection and laser interstitial thermal therapy are effective in the treatment of HH‐related epilepsy, with a tolerable safety profile. Both gelastic and non‐gelastic seizures can be treated, also in patients with a long history of seizures.

**Plain Language Summary:**

This study collected data about 42 patients with HH‐related epilepsies. Endoscopic disconnection and laser therapy are both effective and safe in the treatment of hypothalamic hamartoma‐related epilepsies.

**Key points**

- Endoscopic disconnection and laser interstitial thermal therapy are effective and safe for hypothalamic hamartomas‐related epilepsy treatment.
- Outcome on gelastic and non‐gelastic seizures are similar.
- Patients with a long history of seizures are not prevented from being seizure‐free.
- Central precocious puberty improved after surgery in the 58% of patients.
- A tendency toward improvement is expected for neuropsychological and behavioral disorders following surgery.

## 1 INTRODUCTION

Hypothalamic hamartomas (HH) are rare developmental lesions of non‐neoplastic origin, composed of abnormally arranged neurons and glia, arising from the ventral hypothalamus. HH can be associated with a variety of sign and symptoms, involving the cognitive, behavioral, and endocrinological domains. Gelastic seizures are the HH‐related epilepsy hallmark, even though other type of seizures can be frequently observed such as dacrystic, infantile and epileptic spasms, focal seizures with impaired awareness, atypical absences, tonic, atonic, and generalized tonic–clonic seizures.^1^ Since a high rate of patients (50%–100%) become resistant to medical treatment,^1^ several surgical options have been developed over years, ranging from standard microsurgical procedure (MS), including pterional and interhemispheric approach, to minimally invasive treatments, such as stereotactic radiosurgery (RS), stereotactic radiofrequency thermocoagulation (RF‐TC), endoscopic resective/disconnective surgery (ES) and laser interstitial thermal therapy (LITT).^2^

Despite the high range of the available surgical strategies, the relative low occurrence of HH limits the development of comparative trials, aiming to define the most appropriate option. Consequently, the choice of a specific approach is not univocal so far, mainly depending on available facilities and surgeon’ experience.^3^

In the present study we performed a nation‐based survey, with the aim of investigating the Italian experience on the surgical and radiosurgical treatment of drug‐resistant epilepsy (DRE) due to HH in the period 2011–2021 in six epilepsy surgery centers, and comparing safety and efficacy profiles of the different techniques.

## 2 METHODS

### 2.1 Data collection

The Survey involved the Italian Neurosurgery units that actively collaborates to the Commission of Epilepsy Surgery of the Italian League Against Epilepsy (LICE) (eight centers) and other two centers with consolidated epilepsy surgery programs (four pediatric epilepsy surgery centers and six epilepsy surgery teams in general hospitals or clinical institutions dedicated to clinical neuroscience). In line with national and international recommendations,^4^ only centers providing a presurgical multidisciplinary assessment were considered.

Pseudo‐anonymized data were requested for those patients who underwent a surgical/radiosurgical treatment of HH‐related epilepsy between 2011 and 2021, with at least 12 months of follow‐up. Seizure outcome was defined according to Engel classification of seizure outcome.^5^ A dedicated questionnaire was sent to each Group, asking for: age, age at seizure onset, age at surgery, type of seizures, seizure frequency, type of hamartoma according to Delalande classification,^6^ possible association with behavioral and endocrinological disturbances, type of surgical/radiosurgical treatment and year of the procedure, epilepsy, cognitive, and behavioral outcomes.^7^

Each center sent pseudo‐anonymized data of each patient to M.R. Informed consent for all procedures was obtained.

### 2.2 Statistical analysis

Univariate analysis was performed to assess the risk of post‐operative seizures, categorized in dichotomous variable as favorable (Engel Class I) or unfavorable (Engel Class II to IV). Several explanatory variables were considered for univariate analysis (multivariate analysis was not performed due to the limited sample size). Mann–Whitney or Chi‐squared test were respectively used in order to assess the presence of an association between categorical or numerical variables, as appropriate. *p* < 0.05 were considered statistically significant and all tests were two‐sided. STATA statistical software, version 16 (StataCorp. 2019. Stata Statistical Software: Release 16. College Station, TX: StataCorp LLC) was used for the statistical analysis.

## 3 RESULTS

Six centers out of 10 provided full pseudo‐anonymized information about surgical/radiosurgical procedure on 42 patients, providing dataset concerning the presurgical work‐up and the postsurgical follow‐up (Meyer Children's Hospital in Florence provided two cases, Bambino Gesù Children's hospital in Rome 26, Istituto delle Scienze Neurologiche in Bologna two cases, Carlo Besta Neurological Institute in Milan two cases, Gaslini Children's Hospital in Genoa eight cases and Policlinico Gemelli in Rome two cases). Four centers did not perform surgical/radiosurgical treatment of HH in the considered period.

### 3.1 Presurgical evaluation

All centers performed at least 1‐h scalp video‐EEG in all patients, all but one providing at least 24‐h long‐term monitoring for all patients. Invasive recordings were not performed.

All centers performed at least one preoperative brain‐MR (in one case a 7T study was performed). FDG‐PET was carried out for selected cases in two centers.

All centers provided a complete neuropsychological evaluation (neuropsychological assessment included at least an examination of developmental/cognitive functioning measured by Wechsler Intelligence Scales and the evaluation of affective and behavioral profile by means of self‐report questionnaires and/or clinical observation), based on the Italian league against epilepsy (LICE) position document for adults.^7^ In two centers, all patients had a psychiatric evaluation, in three, it was performed in selected patients only. Endocrinological assessment was performed in all centers. Histological examination was performed in cases undergoing endoscopic disconnection.

Surgical indication was offered after multidisciplinary case conference and procedure selection was based on single‐center expertise and technological availability.

### 3.2 Postsurgical follow‐up

Postoperative evaluations were carried out at least at 6 months and at 1 year in all centers; in five centers, the evaluation was performed also at 3 months after surgery, while in four centers also at 1 month.

In all centers, the postoperative assessment included clinical examination and at least one postoperative brain‐MR. Two centers performed at least one‐hour scalp video‐EEG in selected patients, independently from seizure recurrence. All patients had at least one EEG in the follow‐up.

Post‐operative neuropsychological evaluations were performed in all centers. Two centers assessed the psychiatric profile in all patients, in three centers only in selected cases and in another one it was not performed.

### 3.3 Surgical outcome and trends

Our population consisted on 25 males and 17 females with a median age at surgery of 8.25 years (IQR 9.25).

Twenty‐one of the patients presented with a type 2 hamartoma (50%) (following Delalande Classification), 14 (33%) a type 3, 5 (12%) a type 4 and 2 (5%) a type 1, with an increasing number of operated type 2 hamartoma in the last 3 years.

Age at seizures onset (median 2 years, IQR 0.55–3) and disease duration (median 5.1 years, IQR 2.03–13.15) was not modified over years, even though in the last 2 years (2020 and 2021) only children underwent surgical or radiosurgical therapy.

Forty‐one patients exhibited gelastic seizures, with a daily frequency in 35 (86%), weekly in 5 (12%) and monthly in 1. Thirty‐nine patients suffered from non‐gelastic seizures, with a daily frequency in 21 (54%), weekly in 6 (15%), monthly in 8 (21%) and yearly in 4 (10%).

ES was performed in 30 patients, LITT in 10 and RS in 2. Two patients had two surgeries, one patient was treated with two ESs and another one by means of two LITT.

**FIGURE 1:**
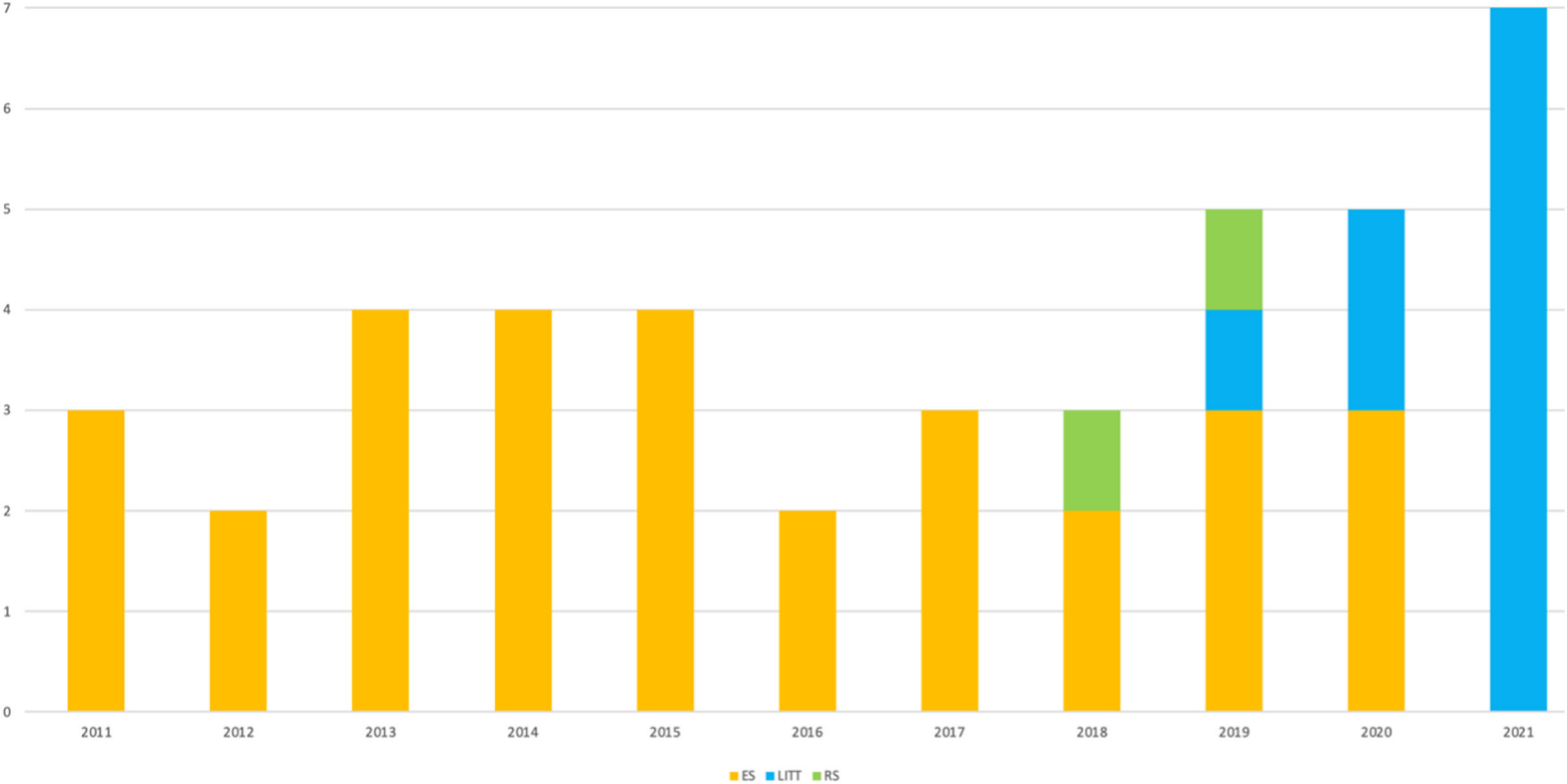
Trend on type of treatment, all the centers combined.

The median number of surgeries per year was 4 (IQR 3–5) with a slight increase between 2019 and 2021 (Figure 1). A rise in LITT procedures was recorded since 2019, together with a reduction in ES, with an increased number of centers involved in the treatment of HH patients.

Engel class I for all types of seizure combined was reached in 23 patients (54.7%). Engel class I was reached in the 65.8% and 66.6% of patients with gelastic and non‐gelastic seizures, respectively. Engel class Ia was reached in the 63.4% and 65.4% of patients with gelastic and non‐gelastic seizures, respectively. All patients but one and 39 out of 42 patients respectively presented with gelastic and non‐gelastic seizures. The three patients presenting with gelastic seizures only are not seizure‐free (2 in Engel class 3 and 1 in Engel Class 4). The patient with isolated non‐gelastic seizures belongs to Engel class 2.

In eight patients (19%) antiseizure medications (ASMs) were stopped following progressive tapering (all of these patients are in Engel class I); in 15 patients (36%) ASMs tapering is ongoing, in four patients (9%) tapering was stopped because of seizures recurrence, while in 15 patients (36%) ASMs were not modified.

Two patients (4.7%) suffered from permanent neurological disorders, namely a slight left hemiparesis in one case and impairment of fixation memory in another one, both following ES.

Twenty‐nine (90.6%) out of 32 patients with a baseline normal behavior resulted stable after surgery, while 3 patients (9.4%) worsened (aggressivity). Seven patients out of 10 with an altered behavioral baseline improved after surgery, while 2 worsened (aggressivity) and one remained stable. Behavioral and neuropsychological improvement have been more frequently in the period 2018–2021.

Two other patients (4.7%) developed a hypothyroidism and hypocorticosurrenalism, respectively.

### 3.4 Univariate analysis on seizure outcome for all seizures type

Two variables were significantly associated with seizure freedom: other than daily non‐gelastic seizure frequency (*p* = 0.01) and the good endocrinological outcome (*p* = 0.001). Despite the absence of significant results, a trend toward association with seizure outcome was recorded for a normal pre‐operative behavioral status and Delalande type 2 HH (Table 1).

**TABLE 1:** Univariate statistical analysis of categorical outcome predictors on seizure freedom, considering outcome for all seizure types together.

Variable	Categories	Engel's class I	Engel's class II‐IV	*p*‐value*
Sex	Male	14	11	0.85
	Female	9	8	
Adult at surgery	Yes	4	5	0.48
	No	19	14	
Interictal EEG epileptic abnormalities	Focal	9	10	0.33
	Multifocal	9	5	
Gelastic	Daily	18	17	
seizure frequency	Other	5	1	0.14
Non gelastic	Daily	8	13	
seizure frequency	Other	14	4	0.01
Pre‐operative Neuropsychological status	Abnormal	7	9	0.20
	Normal	16	9	
Neuropsychological outcome	Improved	8	3	0.41
	Stable	13	11	
	Worse	2	3	
Pre‐operative behavioral status	Altered Normal	3 20	7 12	0.07
Behavioral outcome	Improved	3	4	0.55
	Stable	18	12	
	Worse	2	3	
Developmental milestone	Altered	3	5	0.27
	Normal	20	14	
Psychomotor development outcome	Improved	2	3	0.42
	Stable	16	11	
Pre‐operative endocrinological status	Altered	8	5	0.55
	Normal	15	14	
Endocrinological outcome	Improved	7	0	0.001
	Stable	16	6	
	Worse	0	6	
Neuroimaging delalande class	1, 3, 4	9	12	0.12
	2	14	7	
Type of surgery	ES	18	12	0.24
	LITT	5	5	
	RS	0	2	

* *p*‐values from Chi‐squared test.

Greater age at seizure onset resulted in trend toward associated to seizure‐freedom, although in the absence of significant results (Table 2).

**TABLE 2:** Univariate statistical analysis of numerical outcome predictors on seizure freedom, considering outcome for all seizure types together.

Variable	All patients mean ± SD	All patients median (min–max)	Outcome on seizures		*p*‐value*
			Engel I	Engel II‐IV	
Age at seizure onset	2.73 ± 2.99	2 (0–12)	3.25 ± 3.04	2.10 ± 2.88	0.08
Duration of disease	9.92 ± 11.84	5.1 (0.6–55)	9.39 ± 8.49	10.56 ± 15.18	0.25

* *p*‐values from Mann–Whitney test.

## 4 DISCUSSION

In this study we report the first nationwide survey on the surgical and radiosurgical treatment of patients with HH‐related DRE.

The survey involved 6 Italian epilepsy surgery centers over a period of 11 years. Only two centers (defined as “established”) [(Meyer Hospital (Florence) and Bambino Gesù Children's hospital (Rome)] had experience on HH treatment by ES before 2018. The other four centers (defined as “new”) [“Bellaria” Hospital (Bologna), “Carlo Besta” (Milan), “Gaslini” Hospital (Genoa), and “Gemelli” Hospital (Rome)] engaged in this field since 2018, using different methodologies, namely ES, LITT and RS. We hypothesize that the increased number of centers dedicated to pediatric epilepsy surgery and the earlier referral of these patients^8^ are the main reasons for a positive trend in the volume of DRE‐HH treated patients. LITT is a relatively recent and spreading procedure with promising results in terms of both efficacy and safety. Two new centers started to treat HH patients with LITT without implementing ES, and all the other centers are moving toward the implementation of LITT in the surgical armory.^8^

All centers collected anatomical (with brain‐MR) and electroclinical (with at least 1‐h scalp video EEG, and neuropsychological assessment and endocrinological evaluation) data in the pre‐surgical workup, confirming that scalp video EEG may have a normal interictal profile and a difficult to localize ictal EEG.^1^ Neuropsychological assessment was performed in all patients either at pre‐operative and post‐operative step. Psychiatric evaluation was not homogeneously considered, and standardizing evaluation protocol could overcome this limit, due to the high frequency of such disorders in HH patients.^1^ FDG‐PET is performed in two centers, and this could represent a valuable tool for those patients with a suspicion of an epileptogenic zone extension outside of the HH.

In the post‐surgical follow‐up, all patients underwent brain MR.

Although new techniques have been adopted, the good outcome on seizure did not change over years, either considering gelastic (Figure 2a) and non‐gelastic seizures (Figure 2b). This is in line with a recent meta‐analysis, in which a superiority among ES, LITT and RF‐TC was not demonstrated. On the other hand, each technical solution offers limitations and advantages, so that treatment can be tailored according to single patient features. ES allows to visualize the HH attachment to the third ventricle wall, so that an effective resection/disconnection can be directly controlled. LITT and RF‐TC (namely the stereoelectroencephalography‐guided one) are minimally invasive in terms of extracerebral tissue violation, so that a reduced hospital stay and convalescence period are expected. LITT presents with the advantage of intraoperative lesioning control (due to the use of thermal mapping), while RF‐TC overcomes cost related to LITT, has the unique feature of ictal onset zone mapping, but the limit of a reduced ablated tissue size in comparison to LITT.^9^ Seizure outcome is similar to that of the largest modern series with adequate follow‐up of surgically‐treated HH,^10^ even though in our series outcome on gelastic and non‐gelastic seizures resulted similar. The good outcome also for non‐gelastic seizures and in long‐lasting epilepsies support the hypothesis that epileptogenic zone could frequently be still limited to the HH, despite secondary epileptogenesis.^11^

**FIGURE 2a:**
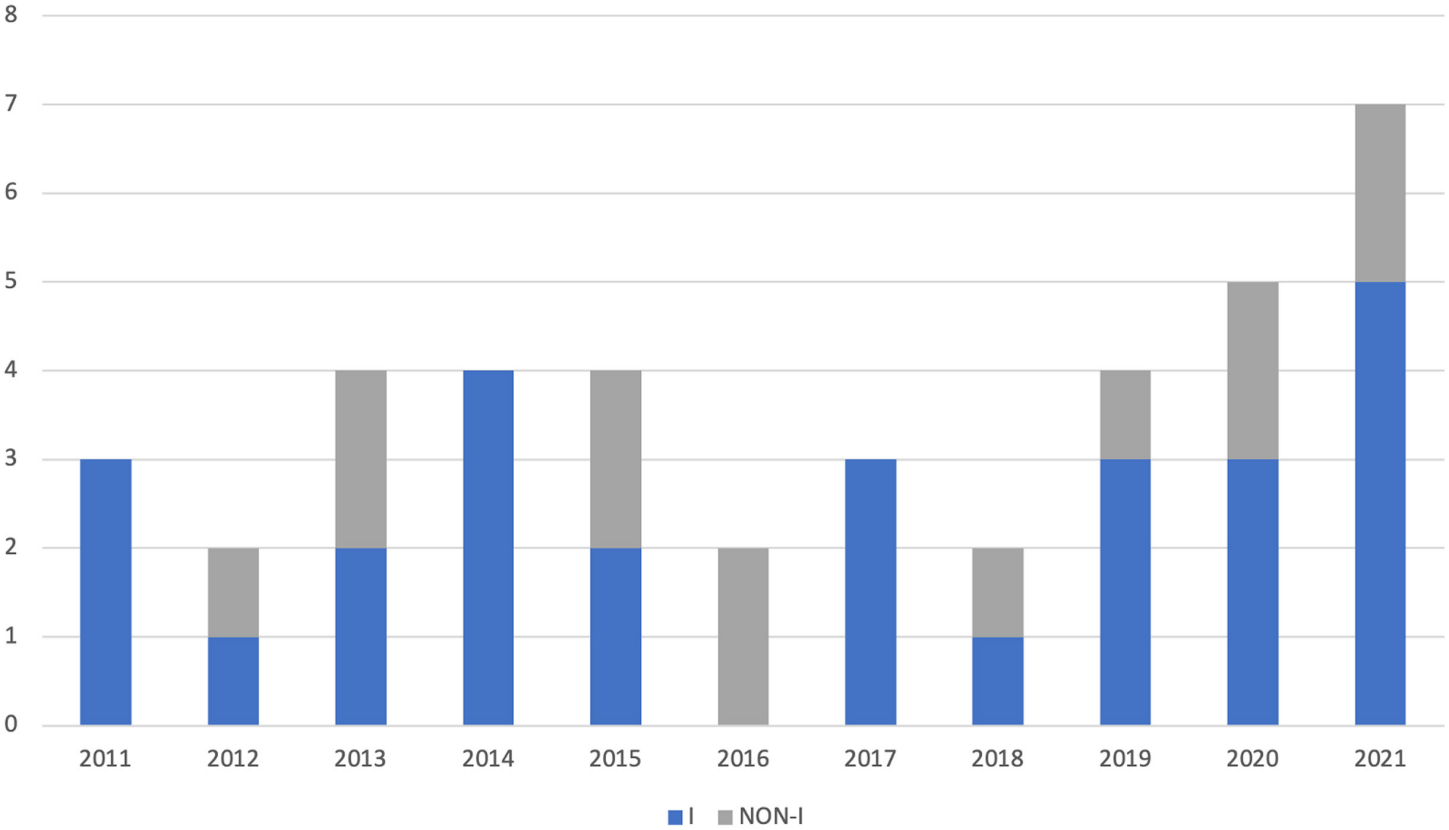
Trend on seizure outcome in patients with gelastic seizures (I = Engel Class I; non‐I = Engel Class II to IV).

**FIGURE 2b:**
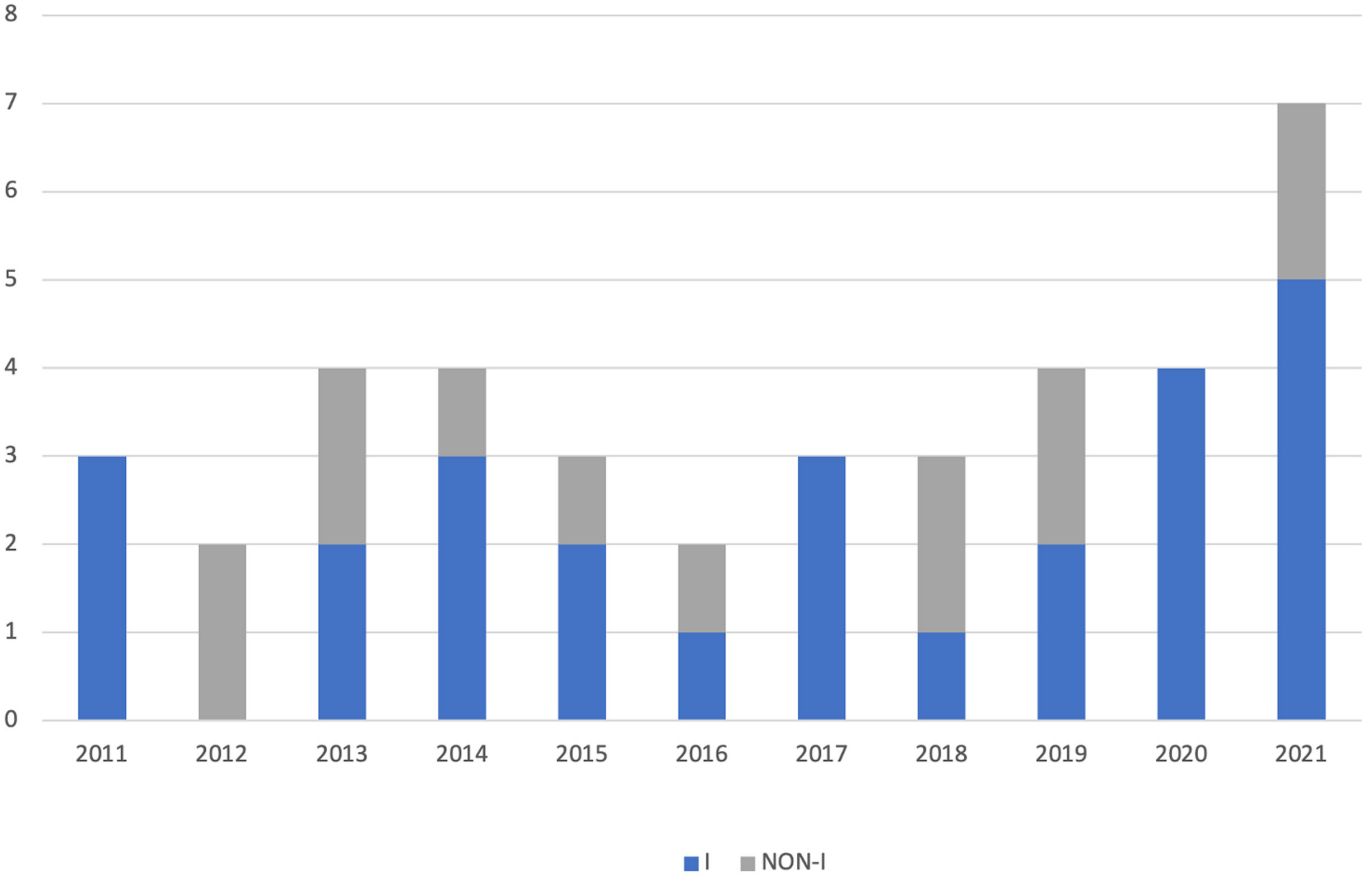
Trend on seizure outcome in patients with non‐gelastic seizures (I = Engel Class I; non‐I = Engel Class II to IV).

Epilepsy duration was not associated with seizure freedom: other studies^10^ claimed that an invasive treatment can be proposed also in patients with a longstanding history of seizures. An older age at seizure onset was associated with seizure freedom for gelastic seizures and in trend toward association for non‐gelastic seizures. A younger age at seizure onset can be symptomatic of a more widespread epileptogenicity, in which an extra‐HH involvement of the epileptogenic zone should be considered. A trend toward association with seizure outcome was also recorded for patients with type 2 HH, in line with other series,^2^ due to the purely intraventricular location. Type 2 was the most frequently treated type of HH, as reported in other series using ES, LITT or RF‐THC^3^, ^10^, ^12^; type I and type III were more frequently reported in microsurgical series^13^, ^14^ and this aspect might reflect a selection bias. Based on the surgical outcome of our report, ES or LITT can be proposed as a first stage treatment for HH‐related epilepsy also in non‐type II HH, without excluding microsurgery after one or more failure of the so‐called “minimally invasive techniques.” Moreover, RF‐TC could represent a valid alternative to expensive techniques, especially in low‐income countries.^10^ RS can still have a role given the best safety profile,^11^ despite limited effectiveness.^15^ In our survey the rate of complications is slightly higher than reported for both ES and LITT,^10^ while RS is confirmed as a safe procedure. Nevertheless, these data should be reviewed following larger series.

Surgery is confirmed to show the tendency toward improvement of neuropsychological, behavioral, and developmental outcomes, in particular for pediatric patients,^16^, ^17^ and following the introduction of LITT in a particular center. Improvement is generally observed in all domains, and this observation might suggest that surgery has a preventive role against encephalopathy.^18^

Patients with central precocious puberty (CPP) and HH, without seizures, are effectively treated by gonadotropin‐releasing hormone administration,^19^ and surgery is considered only in particular conditions.^20^ Ancient series report about the efficacy of microsurgery to treat both CPP and seizures in patients with HH,^21^, ^22^, ^23^ with only one recent consistent study confirming these data, selectively in type I HH.^24^ In our series, CPP improves after surgery in 58% of cases (7/12) (with stability of symptoms in 33%), with all improved patients being also in Engel class I. Surgery resulted successful in treating also type 2, 3, and 4 HH, describing for the first time the efficacy of ES and LITT for CPP resolution in the particular population of patients with HH and seizures.

Another potential benefit of surgery is the discontinuation of ASMs, which occurs in 19% of patients, with a minimum drug withdrawal time of 1.5 year; continuous tapering is ongoing in 35% of patients.^25^

Despite limitations of this study, including its retrospective nature, the limited sample size and data heterogeneity, we confirm that at least two options are effective in the treatment of DRE‐HH patients, with a tolerable safety profile. Both gelastic and non‐gelastic seizures can be treated, also in patients with a long history of seizures. ES and LITT are also effective in treating HH‐associated CPP. Further studies are needed to confirm outcome trends and prognostic factors.

## CONFLICT OF INTEREST STATEMENT

The authors report no relevant conflict of interest. The authors confirm that they have read the journal's position on issues involved in ethical publication and affirm that this report is consistent with those guidelines.
